# The mitral paradox of choice

**DOI:** 10.1007/s12471-019-1246-1

**Published:** 2019-02-20

**Authors:** J. F. Velu, J. J. Piek

**Affiliations:** Amsterdam Medical Centre, Amsterdam, The Netherlands

The implementation of a multidisciplinary heart team for mitral valve disease, ‘the mitral valve heart team’*,* is reported by Heuts and colleagues in the present issue of this journal [1]. This is the first study presenting the implementation of such an approach. In 2016, a total of 158 patients were included and discussed in their mitral valve heart team. Of these patients, 67 were treated surgically, 20 were treated by a transcatheter intervention and 71 were treated conservatively. This study did not aim to document whether the new mitral valve heart team approach would lead to a better clinical outcome. Their future research programme will include a larger number of patients and will focus on a comparison between this new dedicated heart team approach and patients treated by their former heart team approach as a historical control group.

Nowadays, there are many possibilities regarding mitral valve interventions both by surgical and transcatheter means. The number of options is still increasing as more clinical evidence becomes available. A few examples of high-potential mitral interventions are: the Neochord (Neochord Inc., Minneapolis, MN, USA) [2], MitraClip (Abbott Vascular, Menlo Park, CA, USA) [3] and Carillon (Cardiac Dimensions, Kirkland, WA, USA) [4]. Each treatment has its own advantages and disadvantages for a specific patient group. Arising questions are: Which treatments should be offered in a dedicated centre? Which percentage of patients can be treated by a specific technique? How often should we perform this intervention to maintain a skilled performance? And one of the most complex questions: Which treatment is suitable for which patient? This last question goes beyond the morphology of the valve and potential risks of a specific intervention.

The increasing treatment options for mitral valve disease are a positive phenomenon that requires appropriate selection of patients for the most suitable technique. However, the numerous treatment options also have a downside, the so-called paradox of choice [5]. Schwartz [5] states that inherent to the many options to choose from, you will ask yourself afterwards whether you have made the correct decision. According to Schwartz’s approach, appropriate decision-making will involve the following steps. First, figure out your goal or goals. Second, evaluate the importance of each goal. Third, array the options (of treatment). Fourth, evaluate whether each option meets your goals. Fifth, pick the winning option. Finally, evaluate the outcome of your choice that can be used to refine future decision-making.

These phases are also present during a multidisciplinary consultation, such as in the mitral valve heart team. A lot of information is needed to come to the right decision. Not only the anatomy of the mitral valve, visualised with imaging modalities such as echocardiography and computer tomography, is important. Also the personal goal for an individual patient must be taken into account as Gawande states: “But whatever we can offer, our interventions, and the risks and sacrifices they entail, are only justified if they serve the larger aims of a person’s life.” [6].

Altogether, the many treatment options for the mitral valve will not make it easier to decide on the most suitable treatment, either for medical specialists or patients. But the myriad of mitral valve therapies opens up new avenues that were previously unexplored. We are better able to choose the right treatment for a specific pathology, such as treating an annulus dilation by reducing the annulus percutaneously.

Heuts and colleagues [1] provide a novel insight into their strategy of clinical decision-making by means of a dedicated mitral valve heart team. This study focuses on the feasibility of this approach, but larger studies are eagerly awaited that involve both evaluation of the clinical decision-making and documentation of long-term clinical outcome following the selected interventions.
